# Twenty Years of Experience in Juvenile Nasopharyngeal Angiofibroma (JNA) Preoperative Endovascular Embolization: An Effective Procedure with a Low Complications Rate

**DOI:** 10.3390/jcm10173926

**Published:** 2021-08-31

**Authors:** Andrea Giorgianni, Stefano Molinaro, Edoardo Agosti, Alberto Vito Terrana, Francesco Alberto Vizzari, Alberto Daniele Arosio, Giacomo Pietrobon, Luca Volpi, Mario Turri-Zanoni, Giuseppe Craparo, Filippo Piacentino, Paolo Castelnuovo, Fabio Massimo Baruzzi, Maurizio Bignami

**Affiliations:** 1 Neuroradiology Unit, ASST Sette Laghi-Circolo Hospital, 21100 Varese, Italy; andrea.giorgianni@asst-settelaghi.it (A.G.); albertovito.terrana@asst-settelaghi.it (A.V.T.); francescoalberto.vizzari@asst-settelaghi.it (F.A.V.); fabio.baruzzi@asst-settelaghi.it (F.M.B.); 2 Department of Biotechnology and Life Sciences, Division of Neurosurgery, University of Insubria, 21100 Varese, Italy; edoardo_agosti@libero.it; 3 Department of Biotechnology and Life Sciences, Division of Otorhinolaryngology, University of Insubria, 21100 Varese, Italy; albertodaniele.arosio@gmail.com (A.D.A.); tzm@inwind.it (M.T.-Z.); paolo.castelnuovo@me.com (P.C.); 4 Department of Surgical Specialities, Division of Otorhinolaryngology, ASST Sette Laghi-Circolo Hospital, 21100 Varese, Italy; 5 Department of Head and Neck Surgery and Otorhinolaryngology, European Institute of Oncology IRCCS, 20122 Milano, Italy; giacomo.pietrobon@gmail.com; 6 Department of Otorhinolaryngology, ASST Lariana, University of Insubria, 22100 Como, Italy; luca.volpi81@gmail.com (L.V.); bignami67@me.com (M.B.); 7 Department of Surgery, ASST Lariana, University of Insubria, 22100 Como, Italy; 8 Head and Neck Surgery & Forensic Dissection Research Center (HNS&FDRc), Department of Biotechnology and Life Sciences, University of Insubria, 21100 Varese, Italy; 9 Diagnostic and Interventional Neuroradiology Unit, ARNAS Civic Hospital, 90127 Palermo, Italy; gcraparo@yahoo.it; 10 Radiology Unit, ASST Sette Laghi-Circolo Hospital, 21100 Varese, Italy; filippo.piacentino@asst-settelaghi.it

**Keywords:** JNA, embolization, interventional neuroradiology, HNS

## Abstract

Juvenile nasopharyngeal angiofibroma (JNA) is a benign tumor of the nasal cavity that predominantly affects young boys. Surgical removal remains the gold standard for the management of this disease. Preoperative intra-arterial embolization (PIAE) is useful for reductions in intraoperative blood loss and surgical complications. In our series of 79 patients who underwent preoperative embolization from 1999 to 2020, demographics, procedural aspects, surgical management and follow-up outcome were analyzed. Embolization was performed in a similar fashion for all patients, with a superselective microcatheterization of external carotid artery (ECA) feeders and an injection of polyvinyl alcohol (PVA) particles, followed, in some cases, by the deployment of coils . Procedural success was reached in 100% of cases, with no complications such as bleeding or thromboembolic occlusion, and surgical intraoperative blood loss was significantly decreased. In conclusion, PIAE is a safe and effective technique in JNA treatment, minimizing intraoperative bleeding.

## 1. Introduction

Juvenile nasopharyngeal angiofibroma (JNA) is a highly vascularized and histologically benign tumor of the nasal cavity and paranasal sinuses, with aggressive behavior and locally invasive growth patterns [1]. It comprises 0.05% of head and neck tumors and predominantly occurs in young boys, with a mean age of presentation of 15 years [2,3]. The best treatment to date remains surgical removal of the tumor [3]. Preoperative embolization is used for virtually all cases of JNA, resulting in reduction in intraoperative bleeding, occlusion of surgically inaccessible arterial feeding vessels, decreased operative time and improved surgical visualization, identification and protection of adjacent structures [4,5,6]. This results in a significant reduction in overall surgical complications and, despite some reports of safe resection without embolization, it is considered to be the standard of care in most centers [7,8,9]. In this study, we describe our single-center experience in preoperative JNA devascularization with the injection of polyvinyl alcohol (PVA) into major lesion feeders, highlighting the safety and efficacy of this technique. Furthermore, we put emphasis on the detection of external carotid artery (ECA)-internal carotid artery (ICA) anastomoses, defining the main red flags to be considered during preoperative intra-arterial embolization to avoid intraprocedural iatrogenic embolic complications.

## 2. Materials and Methods

### 2.1. Data Collection

The study was performed in compliance with the Helsinki Declaration and with policies approved by the Insubria Board of Ethics. All patients involved in the study signed a consent form to publish their clinical photographs whenever useful.

**Figure 1:**
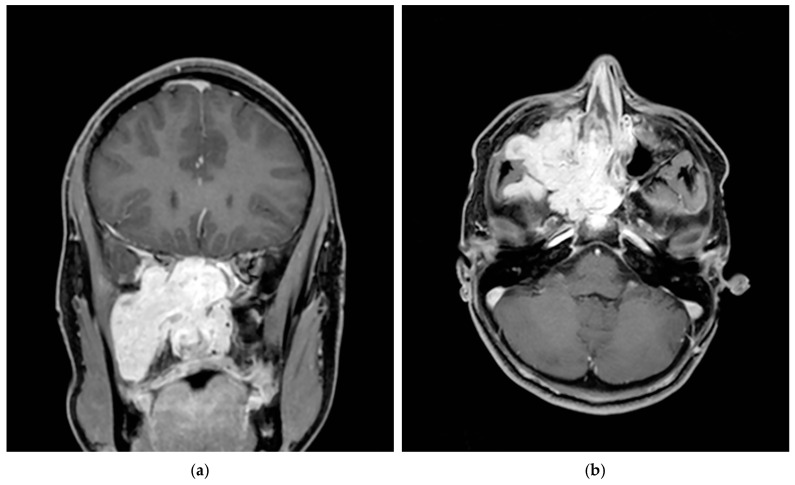
Preprocedural MRI scan (Gd-enhanced T1 Gradient-Echo 3D): coronal (**a**) and axial (**b**) views showing large JNA of right nasopharyngeal mass with expansion of the pterygopalatine fossa and extension into the infratemporal fossa.

We performed a retrospective analysis of 79 patients treated surgically at our Institution for JNA between 1999 and 2020 who underwent PIAE of ECA branches with the sole usage of PVA. Angiographic patterns, Radkowski stage [10], surgical approach, surgical time, blood loss, age and follow-up imaging were also listed in the database in Appendix A. CT and MRI scans were performed in all patients in order to assess Radkowski stage (Figure 1).

The main outcomes considered were the incidence of complications related to embolization and/or surgery, residual disease rate and intraoperative blood loss. 

### 2.2. Endovascular Embolization

**Figure 2:**
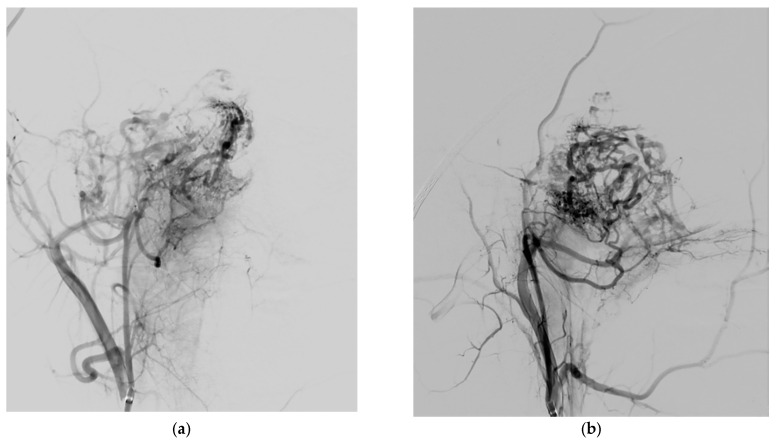
Preoperative DSA: selective catheterization of proximal ECA. Posteroanterior (PA) (**a**) and laterolateral (LL) (**b**) views of the JNA with major feeders from sphenopalatine branches of the distal internal maxillary artery (IMA) and from the ascending pharyngeal artery (APhA).

**Figure 3:**
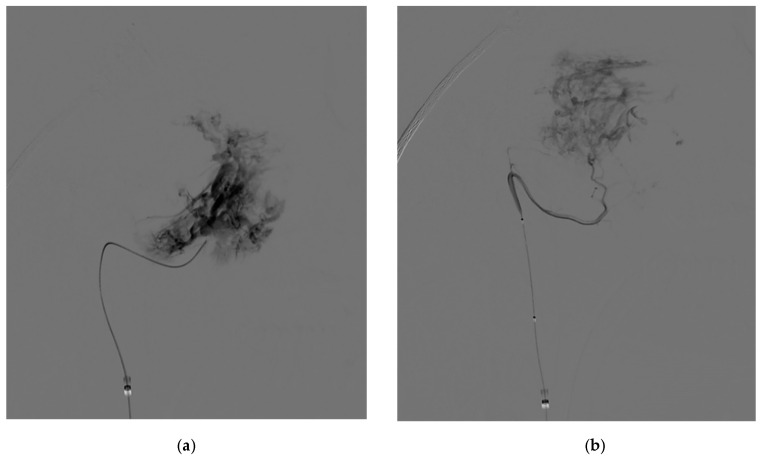
Intraprocedural DSA: PA view of superselective injection of distal IMA (**a**) and APhA (**b**) feeders.

**Figure 4:**
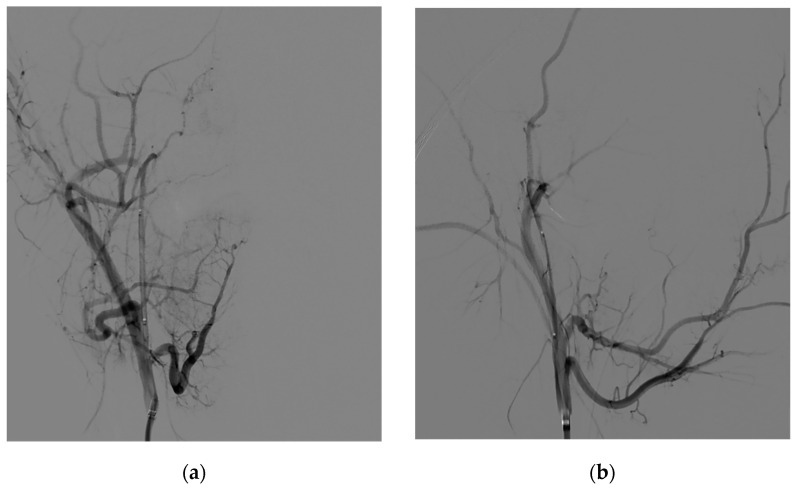
Postprocedural DSA: PA (**a**) and LL (**b**) views of the JNA showing successful embolization of the lesion.

The same approach was performed for every patient, with right groin puncture and placement of 6F femoral sheath, catheterization of internal/external carotid artery (ICA/ECA) and vertebral artery (VA) with angiographic study of their vascular regions (Figure 2), followed by 6F guide catheter (Envoy MPC 90 cm, Cordis) in proximal ECA and superselective catheterization of lesion feeders. Microcatheters used (Rebar 18, Medtronic; SL-10, Stryker) ranged from 0.0165 in to 0.021 in of internal diameters; guidewires used (Traxcess 14, Synchro 10, Synchro 14, Stryker) ranged from 0.010 in to 0.014 in. A control run was then performed from the microcatheter to look for dangerous collaterals and determine the precise position of the distal tip (Figure 3). Embolization was then performed using PVA particles (Contour, Boston Scientific, Marlborough, MA, USA) with different sizes—ranging from 250–355 µm to 500–710 µm—in a slow infusion using blank roadmap visualization to achieve as proper distal penetration as anatomically possible until complete stasis of flow within each feeding vessel was achieved. Adjunctive coil embolization with GDC platinum coil was performed if particle embolization turned out to be incomplete, especially in the case of hypertrophied IMA. At the end of the procedure, control angiography was performed from both ICA and ECA to assess the percentage of tumor feeders embolized. Successful embolization was determined as a lack of contrast in the vascular territory of the embolized vessel (Figure 4).

## 3. Results

In total, 79 patients were included in this series. The mean age was 18 years (range 10–63 years); all of them were male (100%). The most common symptom was epistaxis (55%), followed by nasal obstruction (50%). According to the classification of Radkowski et al., 3/79 (3.8%) type IA, 7/79 (8.9%) type IB, 26/79 (32.9%) type IIA, 7/79 (8.9%) type IIB, 21/79 (26.6%) type IIIA and 15/79 (18.9%) type IIIB tumors were treated. PIAE with PVA intra-arterial injection was performed in all patients. All cases displayed tumor arterial supply from ECA and/or ICA circulations on 2D angiograms, with a total number of arterial tumor feeders embolized in a given session ranging between 1 and 5. 

The technical success of angiography and embolization of almost one big feeder was 100%. Embolization of the JNAs was performed in all cases (79/79) (100%); from distal sphenopalatine branches of the internal maxillary artery in 35/79 cases (44.3%); from ascending pharyngeal artery branches in 20/79 cases (25.3%); from an accessory branch of the middle meningeal artery (MMA) in 7/79 cases (8.9%); from the facial artery and a deep temporal branch of the MMA in 5/79 cases (0.6%); and, in 64/79 cases (81%), procedures were performed under general anesthesia, while the other 15 (18.9%) were performed under conscious sedation.

There was no post-procedural bleeding and there were no thrombo-embolic cerebral ischemic complications in any patient. In no case were there any complications such as vascular dissections, groin hematomas or other complications related to vascular microcatheterization or embolization. Neck pain was experienced by a few patients, and was promptly resolved with analgesic medications. Tumors was removed in all cases within 24 h after embolization. All patients underwent surgery through an endoscopic endonasal approach. All patients were neurologically intact after surgery. Diagnosis of JNA was confirmed histopathologically after surgery. 

Follow-up imaging was predominantly performed with MRI. Residual lesions were identified in 7/78 patients (8.9%). In post-surgical remnant JNA patients, the mean size of the preoperative lesion and the presence of vascular afferent from the ICA was greater than in JNAs, in which gross total resection occurred. Of all the post-surgical remnant JNAs, only 2/79 (2.5%) underwent new surgical treatment. Demographic, clinical and surgical data of the 79 patients are summarized in Table 1.

## 4. Discussion

In this study, we documented an excellent safety profile of PIAE with PVA, reporting no complications directly related to the embolization.

JNA is a rare, benign, vascular lesion of the skull base that affects young adolescent males most commonly between 9 and 19 years of age [3,10]. It is highly aggressive and associated with significant morbidity. Its tendency for skull base erosion, intracranial extension (20% of cases) and high vascularity (vascular component in a fibrous stroma with single endothelial lining) make surgical resection challenging, with a relevant risk for blood loss during resection, post-surgical remnants and lesion recurrence [9]. 

JNA commonly originates in the posterolateral wall of the nasal cavity, near the superior margin of the sphenopalatine foramen, with progressive diffusion to the anterior nasal cavity, maxillary sinus, pterygoid region, infratemporal fossa and middle cranial fossa [11,12,13]. Signs and symptoms are most often related to tumor extension into the nose, leading to nasal obstruction and epistaxis [10,13,14]. Feeding vessels usually arise from the external carotid system via the internal maxillary artery or ascending pharyngeal artery, but can be highly variable, often with heterogeneous vascularization patterns originating from contralateral ECA, petrous and cavernous branches of ICA, such as mandibulo-vidian artery, inferolateral trunk and ECA-ICA anastomosis, such as ethmoid branches of the ophthalmic artery, which are often related to bigger dimensions [15,16,17]. 

Traditionally, the open transfacial approach has been the gold standard for JNA excision [1]. In recent years, the advent of endonasal endoscopic approaches (EEAs) has revolutionized the surgical management of these lesions, reducing JNA post-surgical morbidity and recurrence rates [14]. The main advantages of the endoscopic endonasal route are better magnification of the lesion, the dissection of the surgical planes between the lesion and healthy tissue and better cosmetic outcomes. However, JNA resection can still be complicated by massive hemorrhaging because of a rich vascular supply [14,15]. 

In order to reduce intraoperative bleeding, facilitate surgical lesion removal and improve a patient’s post-operative course, over time, preoperative embolization techniques have been established [18]. The main techniques used for preoperative JNA embolization are endovascular arterial catheterization and direct percutaneous puncture [16,17,18,19].

Pharmacological treatments also have been described to minimize the intraoperative bleeding. Thakar et al. described a significant difference between prepubertal and postpubertal patients in their response to flutamide. Indeed, in postpubertal patients, 6 weeks preoperative may lead to partial tumor regression, facilitating surgical excision and limit morbidity [20].

PIAE is the current most accepted treatment for JNA, minimizing intraoperative bleeding and reducing surgical morbidity [15,17,21,22]. This technique not only reduces the blood supply to the lesion, but the diagnostic preoperative digital subtraction angiography (DSA) highlights the JNA specific vascularization patterns, guiding the surgeon to plan the approach and to delineate lesion areas of increased bleeding risk [15,23]. However, the intra-arterial embolization has some technical limitations, mainly due to the presence of non-embolizable small feeders and to the vascular spasm caused by catheter endovascular manipulation [19]. Furthermore, the presence of ECA-ICA anastomosis directly involved in the vascular supply limits complete JNA devascularization for the risk of inadvertent injection of embolic material into ICA circulation by anterograde crossing from ECA branches through the tumor feeders [16,23,24]. These embolic complications can lead to retinal and cerebral strokes, with iatrogenic blindness and permanent brain damage [24,25,26].

The widely used standard approach for JNA is embolization with particles such as PVA, embospheres (Guerbet Biomedical, Louvres, France) and gelfoam (Upjohn Co., Kalamazoo, MI, USA), all of which have been used successfully for the PIAE of head/neck tumors, as well as in the central nervous system [15,22]. The use of liquid embolic agents (e.g., Onyx), also by percutaneous direct puncture, has been reported to allow for a deeper penetration to tumor capillaries with improved fluoroscopic visibility, as well as a lower risk of catheter adherence [17]. When using PVA, because of its irregular profile (“flakes”), a minimum particle size of more than 150 μm, with a range from 150 to 350 μm, is believed to provide the best compromise between safety (collaterals) and efficient devascularization. As only a temporary occlusion can be achieved, an interval no longer than 7 days between particle embolization and surgery is essential to ensure sufficient devascularization [17,19,27].

## 5. Conclusions

In this retrospective analysis, PIAE has demonstrated itself to be a safe technique (absence of major intra- or periprocedural hemorrhagic or ischemic complications) and, above all, effective in reducing intraoperative bleeding. Additionally, offering improved intraoperative visibility also reduces postoperative JNA residual rates.

## Figures and Tables

**Table 1:** Demographic, clinical and procedural characteristics of the 79 patients.

Variables		Data
Age (years)	Mean	18
	Median	20
	Range	10–63
Symptoms	Epistaxis	55%
	Nasal obstruction	50%
	Rhinolalia	14%
	Headache	12%
	Proptosis	10%
	Diplopia	6%
	Decreased visual acuity	2%
Radkowski classification	Type IA	4%
	Type IB	9%
	Type IIA	33%
	Type IIB	9%
	Type IIIA	26%
	Type IIIB	19%
Intraoperative blood loss (mL)	Mean	784
	Range	40–5200
Surgical time (min)	Mean	217
	Range	95–625
Neuroimaging follow up (months)	Mean	25
	Median	12
	Range	1–127

## Data Availability

Data are available on request due to restrictions, e.g., privacy or ethical reasons.

## Appendix A

**Table A1:** Demographic, procedural, surgical and follow-up data of patients treated in the cohort.

Patient No.	Age	Date of Treatment	Radkowski Staging	Sedation	Selective Embolization	Adjunctive Coils	Blood Loss (mL)	Embo/Surgery Complications	Persistence	Surgery for Recurrence
1	20	20 July 1999	IIb	GA	IMA/APhA	No	500	No	No	No
2	10	18 November 1999	Ia	CS	IMA	No	400	No	No	No
3	43	7 May2001	IIIa	GA	IMA	No	400	No	No	No
4	18	15 May 2001	IIa	CS	IMA	No	400	No	No	No
5	16	20 February 2002	Ia	GA	IMA/APhA	No	400	No	No	No
6	13	28 February 2002	IIIa	GA	IMA/APhA/FA	No	650	No	No	No
7	17	3 June 2002	IIa	CS	IMA	No	200	No	No	No
8	14	29 August 2002	IIIb	GA	IMA/APhA	No	2800	No	Yes (CS)	No
9	31	12 November 2002	IIIa	CS	IMA	No	200	No	No	No
10	19	28 January 2003	IIa	GA	IMA	No	400	No	No	No
11	16	12 March 2003	IIIb	GA	IMA/APhA	No	1500	No	No	No
12	12	19 February 2004	IIIb	GA	IMA/APhA/AM/DT	Yes	800	No	Yes (CS)	Yes (6/12/2005)
13	12	2 February 2005	IIa	GA	IMA	No	450	No	No	No
14	49	14 February 2005	IIa	GA	IMA/APhA	No	200	No	No	No
15	16	21 February 2005	Ia	CS	IMA	No	300	No	No	No
16	13	1 March 2005	IIIa	GA	IMA	No	700	No	Yes (PPF)	No
17	10	14 March 2005	IIIa	GA	IMA/APhA/AM	No	1400	No	No	No
18	29	13 June 2005	IIIa	CS	IMA	No	500	No	No	No
19	36	5 September 2005	IIIb	GA	IMA/APhA/FA	Yes	2500	No	Yes (CS)	No
20	15	29 November 2005	IIIb	GA	IMA/APhA/AM	No	1800	No	Yes (CS)	No
21	12	24 January 2006	IIIa	GA	IMA	No	800	No	No	No
22	19	9 March 2006	IIa	CS	IMA	No	200	No	No	No
23	13	14 December 2006	IIa	GA	IMA/APhA	No	1500	No	No	No
24	13	18 June 2007	IIIa	GA	IMA	No	350	No	No	No
25	15	28 June 2007	IIIa	GA	IMA/AM	No	500	No	No	No
26	17	12 July 2007	IIa	GA	IMA	No	200	No	No	No
27	14	23 October 2007	IIIb	GA	IMA/APhA/FA	Yes	600	No	No	No
28	15	11 June 2008	IIIb	GA	IMA/APhA/AM	No	700	No	No	No
29	39	30 June 2008	IIIb	CS	IMA	No	200	No	No	No
30	20	22 July 2008	IIIb	GA	IMA/APhA/DT	No	5200	No	No	No
31	20	9 September 2008	IIIb	GA	IMA/AM	No	300	No	No	No
32	14	30 September 2008	IIIa	CS	IMA	No	300	No	No	No
33	14	11 November 2008	IIa	GA	IMA/APhA/FA	No	300	No	No	No
34	21	2 December 2008	IIb	GA	IMA/AM	No	700	No	No	No
35	18	17 February 2009	IIb	CS	IMA	No	1200	No	No	No
36	26	16 June 2009	IIIa	GA	IMA/APhA/DT	No	600	No	No	No
37	13	21 January 2010	IIIa	GA	IMA/APhA/AM	No	1000	No	No	No
38	18	25 March 2010	IIb	GA	IMA	No	800	No	No	No
39	13	11 May 2010	IIa	GA	IMA	No	300	No	No	No
40	18	2 June 2010	IIa	GA	IMA/APhA	No	100	No	No	No
41	20	9 November 2010	IIa	GA	IMA	No	1500	No	No	No
42	16	29 November 2011	IIb	GA	IMA/APhA	No	550	No	No	No
43	15	17 April 2012	Ib	GA	IMA/AM	No	250	No	No	No
44	13	22 May 2012	IIIa	GA	IMA/APhA	Yes	600	No	No	No
45	20	19 June 2012	IIa	CS	IMA	No	100	No	No	No
46	14	26 July 2012	IIIa	GA	IMA/APhA/AM	No	800	No	No	No
47	19	23 August 2012	IIa	GA	IMA	No	600	No	No	No
48	17	4 September 2012	IIa	GA	IMA	No	400	No	No	No
49	19	16 October 2012	IIIa	GA	IMA/AM	Yes	400	No	No	No
50	19	8 January 2013	IIa	GA	IMA	No	300	No	No	No
51	13	14 May 2013	IIIa	GA	IMA/APhA/AM	No	200	No	No	No
52	13	19 June 2013	Ib	GA	IMA	No	500	No	No	No
53	13	20 June 2013	IIIa	GA	IMA	No	2000	No	No	No
54	63	24 June 2013	IIa	GA	IMA	No	500	Post-surgical bleeding	No	No
55	11	25 June 2013	IIIb	GA	IMA/APhA	No	1200	No	Yes (CS)	No
56	13	27 August 2013	IIa	GA	IMA	No	800	No	No	No
57	20	16 January 2014	IIa	GA	IMA/AM	No	250	No	No	No
58	16	2 April 2014	IIa	GA	IMA	No	40	No	No	No
59	16	29 September 2014	IIIa	GA	IMA	No	450	No	No	No
60	15	12 November 2014	IIIb	GA	IMA/APhA/AM	No	1750	No	Yes (MCF)	No
61	12	25 February 2015	IIIb	GA	IMA	No	2000	No	No	No
62	15	11 March 2015	IIIb	GA	IMA/APhA/AM	No	2100	No	No	No
63	18	15 July 2015	IIIa	GA	IMA/APhA/AM/DT/FA	No	1500	No	No	No
64	35	20 January 2016	IIIa	GA	IMA/APhA/DT/FA	No	1000	No	No	No
65	13	31 August 2016	IIb	GA	IMA/APhA/AM	No	500	No	No	No
66	12	22 September 2016	Ib	GA	IMA/APhA/AM	No	150	No	No	No
67	16	7 June 2017	IIa	CS	IMA	No	200	No	No	No
68	16	19 July 2017	IIIa	GA	IMA/APhA/AM	Yes	2500	No	No	No
69	13	19 October 2017	IIa	GA	IMA/APhA	No	150	No	No	No
70	14	23 January 2018	Ib	GA	IMA/APhA/AM	Yes	200	No	No	No
71	19	10 July 2018	Ib	CS	IMA	No	100	No	No	No
72	16	16 August 2018	Ib	CS	IMA	No	50	No	No	No
73	12	18 February 2019	IIb	GA	IMA	No	150	No	No	No
74	15	26 June 2019	IIa	GA	IMA/APhA	No	500	No	No	No
75	14	26 September 2019	IIb	CS	IMA	No	300	No	No	No
76	18	28 November 2019	IIb	GA	IMA	No	100	No	No	No
77	20	19 December 2019	IIa	GA	IMA	No	400	No	No	No
78	15	4 May 2020	IIa	GA	IMA/APhA/FA	No	2000	No	No	No
79	13	30 November 2020	IIIb	GA	IMA/APhA	No	2500	No	No	No

CS: cavernous sinus; PPF: pterygopalatine fossa; MCF: middle cranial fossa; GA: general anesthesia; CS: conscious sedation; IMA: internal maxillary artery; APhA: ascending pharyngeal artery; DT: deep temporal branch of MMA; AM: accessory meningeal branch of MMA; FA: facial artery.

## References

[1] Adil Shroff Makhasana J., Kulkarni M.A., Vaze S., Sarosh Shroff A. (2016). Juvenile nasopharyngeal angiofibroma. J. Oral Maxillofac. Pathol..

[2] Overdevest J.B., Amans M.R., Zaki P., Pletcher S.D., El-Sayed I.H. (2018). Patterns of vascularization and surgical morbidity in juvenile nasopharyngeal angiofibroma: A case series, systematic review, and meta-analysis. Head Neck.

[3] Alshaikh N.A., Eleftheriadou A. (2015). Juvenile nasopharyngeal angiofibroma staging: An overview. Ear. Nose Throat J..

[4] Ballah D., Rabinowitz D., Vossough A., Rickert S., Dunham B., Kazahaya K., Cahill A.M. (2013). Preoperative angiography and external carotid artery embolization of juvenile nasopharyngeal angiofibromas in a tertiary referral paediatric centre. Clin. Radiol..

[5] Maroda A.J., Beckmann N.A., Sheyn A.M., Elijovich L., Michael L.M., DiNitto J.M., Rangarajan S.V. (2020). Trimodal embolization of juvenile nasopharyngeal angiofibroma with intracranial extension. Int. J. Pediatr. Otorhinolaryngol..

[6] Lutz J., Holtmannspötter M., Flatz W., Meier-Bender A., Berghaus A., Brückmann H., Zengel P. (2016). Preoperative embolization to improve the surgical management and outcome of juvenile nasopharyngeal angiofibroma (JNA) in a single center: 10-year experience. Clin. Neuroradiol..

[7] Rosenbaum-Halevi D., Lopez-Rivera V., Turkmani A., Sanzgiri A., Zeineddine U.H., Luong A., Roc P. (2020). A safer endovascular technique for pre-operative embolization of juvenile nasopharyngeal angiofibroma: Avoiding the pitfalls of external carotid artery—Internal carotid artery anastomoses. J. Cerebrovasc. Endovasc. Neurosurg..

[8] Leong S.C. (2013). A systematic review of surgical outcomes for advanced juvenile nasopharyngeal angiofibroma with intracranial involvement. Laryngoscope.

[9] López F., Triantafyllou A., Snyderman C.H., Hunt J.L., Suárez C., Lund V.J., Strojan P., Saba N.F., Nixon I.J., Devaney K.O. (2017). Nasal juvenile angiofibroma: Current perspectives with emphasis on management. Head Neck.

[10] Radkowski D., McGill T., Healy G.B., Ohlms L., Jones D.T. (1996). Angiofibroma. Changes in staging and treatment. Arch. Otolaryngol. Head Neck Surg..

[11] Nicolai P., Schreiber A., Bolzoni Villaret A. (2012). Juvenile angiofibroma: Evolution of management. Int. J. Pediatr..

[12] Rowan N.R., Zwagerman N.T., Heft-Neal M.E., Gardner P.A., Snyderman C.H. (2017). Juvenile nasal angiofibromas: A comparison of modern staging systems in an endoscopic era. J. Neurol. Surg. B Skull Base.

[13] Carrillo J.F., Maldonado F., Albores O., Ramírez-Ortega M.M., Oñate-Ocaña L.F. (2008). Juvenile nasopharyngeal angiofibroma: Clinical factors associated with recurrence, and proposal of a staging system. J. Surg. Oncol..

[14] Snyderman C.H., Pant H., Carrau R.L., Gardner P. (2010). A new endoscopic staging system for angiofibromas. Arch. Otolaryngol. Head Neck Surg..

[15] Gargula S., Saint-Maurice J.P., Labeyrie M.A., Eliezer M., Jourdaine C., Kania R., Wassef M., Adle-Biassette H., Houdart E., Herman P. (2021). Embolization of internal carotid artery branches in juvenile nasopharyngeal angiofibroma. Laryngoscope.

[16] Santos-Franco J.A., Lee A., Campos-Navarro L.A., Tenorio-Sánchez J., Zenteno M., Osorio-Alvarado A.R. (2012). Bilateral non-superselective embolization with particles under transient occlusion of the internal carotid artery in the management of juvenile nasopharyngeal angiofibroma: Technical note. Vasc. Endovasc. Surg..

[17] Lv M.M., Fan X.D., Su L.X., Chen D. (2013). Preoperative direct puncture embolization of advanced juvenile nasopharyngeal angiofibroma in combination with transarterial embolization: An analysis of 22 consecutive patients. Cardiovasc. Interv. Radiol..

[18] Hackman T., Snyderman C.H., Carrau R., Vescan A., Kassam A. (2009). Juvenile nasopharyngeal angiofibroma: The expanded endonasal approach. Am. J. Rhinol. Allergy.

[19] Petruson K., Rodriguez-Catarino M., Petruson B., Finizia C. (2002). Juvenile nasopharyngeal angiofibroma: Long-term results in preoperative embolized and non-embolized patients. Acta Otolaryngol..

[20] Thakar A., Gupta G., Bhalla A.S., Jain V., Sharma S.C., Sharma R., Bahadur S., Deka R.C. (2011). Adjuvant therapy with flutamide for presurgical volume reduction in juvenile nasopharyngeal angiofibroma. Head Neck.

[21] Borota L., Mahmoud E., Nyberg C., Ekberg T. (2015). Combined percutaneous and transarterial devascularization of juvenile nasopharyngeal angiofibroma with protection of internal carotid artery : A modification of the technique. Interv. Neuroradiol..

[22] Narayanan T., Shilpee J., Sharma B. (2017). Imaging in Juvenile Nasopharyngeal Angiofibroma : Clinical Significance of Ramharan and Chopstick Sign. Indian J. Otolaryngol. Head Neck Surg..

[23] Gao M., Gemmete J.J., Chaudhary N. (2013). A comparison of particulate and onyx embolization in preoperative devascularization of juvenile nasopharyngeal angiofibromas. Neuroradiology.

[24] Trivedi M., Desai R.J., Potdar N.A., Shinde C.A., Ukirde V., Bhuta M., Gopinathan Nair A. (2015). Vision loss due to central retinal artery occlusion following embolization in a case of a giant juvenile nasopharyngeal angiofibroma. J. Craniofac. Surg..

[25] Vazquez A., Shukla P.A., Choudhry O.J., Gandhi C.D., Liu J.K., Anderson Eloy J. (2013). Geometric alopecia after preoperative angioembolization of juvenile nasopharyngeal angiofibroma. Allergy Rhinol..

[26] Tawfik K.O., Harmon J.J., Walters Z., Samy R., de Alarcon A., Stevens S.M., Abruzzo T. (2018). Facial palsy following embolization of a juvenile nasopharyngeal angiofibroma. Ann. Otol. Rhinol. Laryngol..

[27] Safadi A., Schreiber A., Fliss D.M., Nicolai P. (2018). Juvenile angiofibroma: Current management strategies. J. Neurol. Surg. B Skull Base.

